# An Extreme Learning Machine Based on Artificial Immune System

**DOI:** 10.1155/2018/3635845

**Published:** 2018-06-25

**Authors:** Hui-yuan Tian, Shi-jian Li, Tian-qi Wu, Min Yao

**Affiliations:** School of Computer Science and Technology, Zhejiang University, Hangzhou, China

## Abstract

Extreme learning machine algorithm proposed in recent years has been widely used in many fields due to its fast training speed and good generalization performance. Unlike the traditional neural network, the ELM algorithm greatly improves the training speed by randomly generating the relevant parameters of the input layer and the hidden layer. However, due to the randomly generated parameters, some generated “bad” parameters may be introduced to bring negative effect on the final generalization ability. To overcome such drawback, this paper combines the artificial immune system (AIS) with ELM, namely, AIS-ELM. With the help of AIS's global search and good convergence, the randomly generated parameters of ELM are optimized effectively and efficiently to achieve a better generalization performance. To evaluate the performance of AIS-ELM, this paper compares it with relevant algorithms on several benchmark datasets. The experimental results reveal that our proposed algorithm can always achieve superior performance.

## 1. Introduction

In recent years, many computational intelligence techniques, such as neural networks and support vector machines (SVMs) [1], have been widely used in many real-world applications. However, those algorithms face some defects such as slow learning speed, trivial human intervention, and poor computational scalability.

Recently, to solve the drawbacks mentioned above, Huang et al. [2–5] proposed a new method named extreme learning machine (ELM) which has attracted ever-growing research attention. In contrast to the traditional neural networks such as BP [6], ELM is a tuning-free algorithm with fast learning speed by randomly generating input weights and hidden biases. With the help of least square method and Moore-Penrose generalized inverse, the ELM is transferred as a linear learning system. In addition, ELM is theoretically proved to have a good generalization performance with least human intervention. Therefore, ELM is widely used in many fields [5]. For example, Chaturvedi et al. [7] extended the extreme learning machine (ELM) paradigm to a novel framework that exploits the features of both Bayesian networks and fuzzy recurrent neural networks to perform subjectivity detection. Gastaldo et al. [8] addressed the specific role played by feature mapping in ELM. Cambria et al. [9] explored how the high generalization performance, low computational complexity, and fast learning speed of extreme learning machines can be exploited to perform analogical reasoning in a vector space model of affective common-sense knowledge. Recently Ragusa et al. [10] tackled the implementation of single hidden layer feedforward neural networks (SLFNs), based on hard-limit activation functions, on reconfigurable devices.

It is known that an appropriate selection of initial weight sets is very vital for training a neural network model [11]. There is a strong correlation between the final solution and the initial weight. However, due to the random determination of some learning parameters, some nonoptimal parameter may be introduced to the model [5], which may put negative impact on the final performance. To solve such a drawback, many relative works have been proposed in the past ten years. A straightforward way is to combine evolutionary methods with ELM [12]. For instance, Zhu et al. [13] utilized differential evolutionary algorithm (DE) to optimize ELM's generated parameters to achieve better performance. In [14], Xue et al. combined genetic algorithm (GA), ELM, and ensemble learning to get a better and stable result. Rather than using GA or DE method, Sarasw athi et al. presented a PSO driven ELM [15], combining with Integer Coded Genetic Algorithm (ICGA) to solve gene selection and cancer classification. In [16], Cao et al. proposed an improved learning algorithm named self-adaptive evolutionary extreme learning machine (SaE-ELM). Similarly, Wu et al. presented a novel algorithm named dolphin swarm algorithm extreme learning machine (DSA-ELM) [17] to solve optimization problems.

However, all the above evolutionary algorithms have different search efficiency to optimize the problem. There is still much space to improve. For example, it is one of the biggest challenges in ELM that some nonoptimal parameters may be introduced to ELM algorithm due to the random generation of parameters. To overcome that challenge, in this paper we propose a new extreme method named artificial immune system extreme learning machine (AIS-ELM). Because artificial immune system (AIS) [18–20] has global search ability [21] and good convergence [22], it can solve some difficulties like slow convergence, getting stuck in local minima, etc. Therefore, we use AIS to optimize ELM to get a better initial weight sets capable of avoiding the training process falling into the local optimum. The original version and preliminary results of this paper's method were proposed by us in ELM2017 [23]. In this paper we have revised the original formulas, compared the AIS-ELM with more algorithms and added new expressions, regression validation and more datasets.

The rest of the paper is arranged as follows. Sections 2 and 3 briefly describe the traditional ELM and AIS methods.  Section 4 proposes the detailed description of AIS-ELM.  Section 5 carries out corresponding experiment: AIS-ELM algorithm is compared with traditional ELM, PSO-ELM, SaE-ELM, and DSA-ELM on five regression problems and eight classification benchmark problems obtained from the UCI Machine Learning Repository [24]; the training times between AIS-ELM and BP and SVM and traditional ELM are compared on three benchmark classification problems. The last section gives a conclusion of this paper.

## 2. Extreme Learning Machine

This section will introduce the extreme learning machine [2–5] proposed by Professor Huang. ELM is developed from a single hidden layer feedforward network and is extended to a generalized single hidden layer feedforward network. Compared to other conventional learning algorithms, the extreme learning algorithm's advantage is that the nodes of the single hidden layer feedforward network need not be adjusted.

Compared with the traditional learning algorithm, the extreme learning machine not only has the smaller error but can reach the smallest norm of weights [5]; because the hidden layer need not to be adjusted in the limit learning machine algorithm, the output weight matrix can be solved by the least squares method.

For *N* arbitrary training samples {(**x**_*i*_, **t**_*i*_)}_*i*=1_^*N*^, where **x**_**i**_ = [x_*i*1_, x_*i*2_,…, x_in_]^*T*^ ∈ **R**^n^ and **t**_**i**_ = [t_*i*1_, *t*_*i*2_,…, *t*_*im*_]^*T*^ ∈ **R**^*m*^, and given activation function *g*(*x*), the standard mathematical model of SLFNs with $\tilde{N}$ hidden nodes is modeled as follows:

(1) $$\begin{array}{c} \overset{\tilde{N}}{\underset{i=1}{\sum }}\beta_{i}g\left(\;w_{i}\cdot x_{j}+b_{i}\;\right)=o_{j},\;j=1,\ldots ,N \end{array}$$

where **w**_*i*_ = [*w*_*i*1_, *w*_*i*2_,…*w*_*in*_]^*T*^ is the weight vector connecting the input neurons and *i*th hidden neuron, *β*_*i*_ = [*β*_*i*1_, *β*_*i*2_,…*β*_*im*_]^*T*^ is the weight vector connecting the *i*th  hidden neuron and the output neurons, and *b*_*i*_ is the threshold of the *i*th hidden neuron.

That standard SLFNs with $\tilde{N}$ hidden neurons given activation function *g*(*x*) can approximate these *N* samples with zero error which means that

(2) $$\begin{array}{c} \overset{N}{\underset{j=1}{\sum }}\left\|\;o_{j}-t_{j}\;\right\|=0 \end{array}$$

There exist *β*_*i*_, **w**_*i*_, and *b*_*i*_ such that

(3) $$\begin{array}{c} \overset{\tilde{N}}{\underset{i=1}{\sum }}\beta_{i}g\left(\;w_{i}\cdot x_{j}+b_{i}\;\right)=t_{j},\;j=1,\ldots ,N \end{array}$$

The above N equations can be written compactly as

(4) $$\begin{array}{c} H\beta =T \end{array}$$

where

(5) H=⋮hxN=⋯gwN~·x1+bN~⋮⋯⋮gw1·xN+b1⋯gwN~·xN+bN~N×N~

(6) β=⋮βN~TN~×m,

Here, **H** is called the hidden layer output matrix [3].The column of **H** is the *i*th hidden node's output vector with respect to inputs **x**_1_, **x**_2_,…**x**_*N*_ and the *j*th row of H is the output vector of the hidden layer with respect to **x**_*j*_. Then the vector **β** (connecting the hidden layer with the output layer) is estimated using the Moore-Penrose generalized inverse of the matrix **H**:

(7) $$\begin{array}{c} \hat{\beta }=H^{†}T \end{array}$$

ELM algorithm can be summarized as shown in Algorithm 1.

## 3. Artificial Immune System

A relatively new area of bioinspired computing is Artificial Immune Systems (AIS). It is inspired by biological models of the natural immune system which has the properties of diversity, distributed computation, dynamic learning, error tolerance, adaptation, and self-monitoring. AIS can be applied to many domains [19] in principle for the reason that it is a general framework for a distributed adaptive system. This section will introduce the AIS in three aspects. Firstly, the clonal selection algorithm is briefly described in Section 3.1. Secondly, Section 3.2 introduces the mathematical model of BCA. Finally, the mathematical model describing the interaction of Antigen-Antibody is represented in Section 3.3.

### 3.1. Mathematical Model of BCA

Each B cell is modeled as binary strings of fixed length *l* for simplicity of calculation. One of the most important design choices in developing an Artificial Immune Systems algorithm is similarity measure or matching rule [25], and it is closely coupled to the encoding scheme. Hamming distance and edit distance are the obvious approximate matching rules.

**Figure 1:**
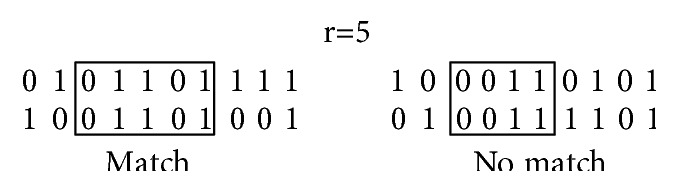
Matching under the rule of* r-contiguous bits*. In this example, *r* = 5, so the left is matching while the right is not.

However, there is a more immunologically plausible rule, called* r-contiguous bits* [26]: two strings will match if they have r-contiguous bits in common (see Figure 1). The value *r* is a threshold which serves as indication of the size of the subset of strings that a single string can match. For example, if *r* = *l*, the matching is completely special; i.e., the string will match only a single string (itself), but if *r* = 0, the matching is absolutely general; that is, the string will match every single string of length *l*.

Besides, in the algorithm a contiguous region hypermutation operator [27] is used and its form is

(8) fT=1L2∑n=1 a∑m=bL−11−rm+1−n−krk+∑n=1an1−rL+1−n−krk

where *f*_*T*_ is the probability of transition from zero to some number *T*  (0 ≤ *T* ≤ 2^*L*^ − 1); *L* is the length of the binary string; *a* is the bit position of the first “flip” bit starting from the most significant bit; *b* is the bit position of the last “flip” bit starting from the most significant bit; *k* is the number of bits that must be flipped to mutate from 0 to *T*. *r* is the mutation probability of a bit given a contiguous region. {*L*, *a*, *b*, *k*, *T*} ∈ *ℤ*^+^; 0 ≤ *T* ≤ 2^*L*^ − 1; *a* ≤ *b* ≤ *L*; {*f*_*T*_, *r*} ∈ *ℝ*; 0 ≤ r ≤ 1

### 3.2. Clonal Selection Theory

The clonal selection theory (CST) [28] is used to explain how the adaptive immune system responds to an antigenic stimulus basically. It establishes the theory that only cells that are capable of recognizing an antigen will proliferate, while those that are incapable of doing so will be eliminated.

Both T cells and B cells can operate clonal selection. In the case of B cells, when the antigen receptors bind with an antigen, B cells begin to clone themselves and undergo somatic hypermutation to introduce diversity into the B cell population. After that B cells become activated and differentiate into plasma or memory cells. Plasma cells produce numerous antigen-specific antibodies leading to the removal of the antigen in a successful immune response. Memory cells remain within the host and promote a rapid secondary response when encountering the same (or similar) antigen. This is the operation of acquired immunity [19].

The B Cell Algorithm (BCA) as a simple clonal selection method was introduced in [22]. An outline of BCA is shown in Algorithm 2.

### 3.3. Shape Space

An abstract model describing the interaction of Antigen-Antibody is introduced by Perelson & Oster [29]. In this model, it is assumed that the characteristics of the antibody receptor (combined region) associated with antigen binding can be described by specifying a total of L shape parameters. It is also assumed that the same L parameters can be used to describe the antigen. These L parameters are incorporated into the vector, and the antibody receptor and antigenic determinant are described as Ab and Ag points, respectively, in an L–Euclidean shape space. Each molecule can be considered as a point in the L-dimensional real space mathematically and the affinity of Ag-Ab is related to the reciprocal of the Euclidean distance between them.

It is assumed that the antibody is capable of binding to any antigenic complement in the distance *ε* (stimulus region). Each *N* dimensional ball of radius *ε* takes up a volume *c*_*N*_*ε*^*N*^, where *c*_*N*_ is a constant which depends upon the dimensionality of N (for arbitrary *N*, *c*_*N*_ = 2*π*^*N*/2^/*N*Γ(*N*/2), where Γ(·) is the Gamma function). If there are a total of *N*_*A*b_ antibodies, its total coverage volume would not be greater than *N*_*A*b_*c*_*N*_*ε*^*N*^ since balls would overlap. Let us assume *V* is an n-dimensional cube with edge length R. The total volume of *V* is then *R*^*N*^.

The goal is to maximize the coverage of antibody which can make the immune approach more reliable. Then the following equation must come into existence:

(9) $$\begin{array}{c} N_{Ab}c_{N}\varepsilon^{N}\geq R^{N} \end{array}$$

Therefore, the range of *N*_*A*b_ is as follows:

(10) $$\begin{array}{c} N_{Ab}\geq \frac{1}{c_{N}}\cdot {\left(\;\frac{R}{\varepsilon }\;\right)}^{N} \end{array}$$

where 0 < *ε* < 1.

## 4. Proposed Extreme Learning Machine Based on Artificial Immune System

This section proposes an Extreme learning machine based on artificial immune system, namely, AIS-ELM. Traditional ELM algorithm randomly generates input weights and hidden biases, and among them there may be some sets of nonoptimal input weights and hidden biases. It is necessary to optimize these nonoptimal input weights and hidden biases. Two methods can be used to solve this problem. One is to increase hidden neurons which is time-consuming and may not get a good result. The other is to optimize the input weights and hidden biases.

This paper combines AIS with ELM to optimize the input weights and hidden biases. AIS-ELM has three main phases: clone phase, mutation phase, and substitution phase. After the three phases, an optimal antibody will be produced. And the performance of ELM will be improved if the optimal antibody is used as the input weights and hidden biases.

One set of input weights and hidden biases are modeled by an antibody; the *ith* antibody is represented by

(11) Abi=a11,a12,…,a1n,a21,a22,…,a2n,aN~1,aN~2,…,aN~n,b1,b2,…,bN~

where *i* = 1,2,…*N*, *N* is the number of training data and the number of population members. $\tilde{N}$ is the number of hidden nodes and *n* is the dimension of input samples. a_*ij*_(*j* = 1,2,…, *n*) are the input weights. *b*_*i*_ are the hidden biases. The initial values of a_*ij*_ and *b*_*i*_ are randomly generated within the range of [-1,1]. Then we calculate each antibody's fitness *Fit*_**Ab**_*i*__(*i* = 1,2,…, *N*) according to the following equation with the validation data {(**x**_*j*_, **t**_*j*_)}_*j*=1_^*V*^.

(12) $$\begin{array}{c} Fit\left(\;Ab_{i}\;\right)=\sqrt{\frac{\sum_{j=1}^{V}||\sum_{i=1}^{\tilde{N}}{\left\|\beta_{i}g\left(a_{i}\cdot x_{j}+b_{i}\right)-t_{j}\right\|}_{2}^{2}}{V}} \end{array}$$

where **T** = [**t**_1_, **t**_2_,…,**t**_*V*_]^*T*^ is the validation data. The reason for using validation data instead of training data is to alleviate possible overfitting. The corresponding output weights β are computed by using the MP generalized inverse by (7).

The clone section creates a clone pool having* N-*1 clonal antibodies **Ac**_**ij**_(*j* = 1,2,…, *N*-1) for every antibody **b**_*i*_, and each clonal antibody is identical to the original antibody, i.e., **Ac**_**ij**_ = **Ab**_**i**_  (*j* = 1,2,…, *N*-1).

In the mutation procedure, each clonal antibody **Ac**_**ij**_ in the clone pool is mutated by the following formula:

(13) $$\begin{array}{c} Ac_{ij}=Ab_{i}\left(\;1+P_{mutation}\;\right) \end{array}$$

where *j* = 1,2,…, *N*-1 and *P*_*mutation*_ is the mutation probability of the clonal antibody.

(14) $$\begin{array}{c} P_{mutation}={\left(\;-1\;\right)}^{j}\cdot j\cdot f_{T}^{\prime }\cdot Fit_{Ab_{i}}\cdot \varepsilon \end{array}$$

Where the following holds.

(1) (−1)^*j*^ · *j* avoids the situation in which the directions of mutation are the same and the result falls into local optimal.

(2) *f*_*T*_′ is as follows:

(15) fT′=1N′2∑y=1a∑x=bN′−11−rx+1−y−krk+∑y=1ay1−rN′+1−y−krk

The above equation is an application of (8), where *N*′ is the total elements of the antibody and $N^{\prime }=\tilde{N}(n+1)$; *f*_*T*_′ is the probability of transition from zero to some number *T*  (0 ≤ *T* ≤ 2^*N*′^ − 1); *a* is the bit position of the first “on” bit starting from the most significant bit; *b* is the bit position of the last “on” bit starting from the most significant bit; *k* is the number of bits that must be flipped to mutate from 0 to *T*; *r* is the mutation probability of a bit given a contiguous region.

(16) N′,a,b,k,T∈Z+;0≤T≤2N′−1;  a≤b≤N′;fT′,r∈R;⍈0≤r≤1.

(3) **Fit**_**Ab**_*i*__ is used to adjust the range of mutation. The smaller the value of fitness is, the smaller the error is, so the requirement of mutation changes is tinier. On the other hand, the greater the value of fitness is, the bigger the need for mutation changes will be.

(4) *ε*  (0 < *ε* < 1) is stimulus region in which the antibody is capable of binding to any antigenic complement [29].

The substitution phase is to calculate each clonal antibody's fitness **Fit**_**Ac**_*ij*__(*j* = 1,2,…, *N*-1) in the clone pool, and to compare **Fit**_**Ac**_*ij*__ with the cloned antibody's fitness **Fit**_**Ab**_*i*__. If **Fit**_**Ac**_*ij*__ is smaller than **Fit**_**Ab**_*i*__, corresponding fitness and antibody will be replaced. For instance, if *i* = 1, *j* = 1, and *Fit*_**Ab**_1__ < *Fit*_**Ac**_11__, it is necessary to replace **Ab**_1_ and *Fit*_**Ab**_1__ with **Ac**_11_ and *Fit*_**Ac**_11__. After this iterative process, the antibody population evolves forward global optimization. Then an antibody with minimal fitness which indicates smallest error is the optimal antibody.

In the above process, our algorithm uses the clonal selection principle to ensure diversity which has been proved by De Castro et al. [30]. In addition, the ELM is optimized by BCA to get a better convergence which has been proved by Clark et al. [22] through an exact Markov chain model. Besides, using **Fit**_**Ab**_*i*__ to adjust the mutation matches up the theory of immune network. Last but not least, the requirement of shape space is also satisfied.

In the specific experiment process, a number of other algorithms have to be compared, so the input data **z** should be normalized to ensure fairness.

(17) $$\begin{array}{c} z^{*}=\frac{z-z_{min}}{z_{max}-z_{min}} \end{array}$$

Then the stop criterion is as follows:

(18) $$\begin{array}{c} SC=1-\sqrt{{\overset{-}{z}}^{T}\overset{-}{z}} \end{array}$$

where $\overset{-}{z}$ is the mean of the group of z.

(19) $$\begin{array}{c} \overset{-}{z}=\frac{1}{N}\overset{n}{\underset{i=1}{\sum }}{z_{i}}^{*} \end{array}$$

All in all, the AIS-ELM have three parts. The first part is initialization including input data, normalization, and set parameters. The second part applies AIS to ELM. After cloning, mutation, and substitution phase, an optimal antibody meeting the requirements is acquired. Then the antibody can be used as the input weights and hidden biases in ELM. The AIS-ELM is presented in Algorithm 3.

## 5. Performance Verification

In this section, AIS-ELM is compared with DS-ELM, PSO-ELM, SaE-ELM, traditional ELM, SVM, and BP. The experiments are divided into two parts. In the first part, the first five algorithms are tested on eight benchmark classification problems; next we compare AIS-ELM with SVM, BP, and traditional ELM on training time on three benchmark classification problems. In the second part, five benchmark regression problems are carried out. The experimental environment is MATLAB R2014b running on a windows pc with Intel 2.7 GHz CPU and 8GB RAM.

All the inputs have been normalized into the range [-1,1] for fairness. The number of hidden neurons depends on different problems and it will be listed in specific experiment. Besides, the parameters for AIS-ELM are set as follows: *a* = 10, *b* = 50, *ε* = 0.1, *k* = 5, *r* = 0.2.

### 5.1. Classification

In this subsection, five algorithms' performances on eight benchmark classification problems are evaluated. The eight datasets are Ecoli, Pima Indians Diabetes (Diabetes), Epileptic Seizure, Iris, Heart Disease, Glass Identification (Glass), Image Segmentation (Image), and Statlog (Satellite), respectively. The detailed description of the eight datasets is listed in Table 1.

Attributes of all the dataset have been normalized to [-1, 1], and the output is the training time, testing accuracy's mean and variance. A 20-fold cross validation method is taken to get the average of 20 repeated experiments to minimize the error. The whole dataset is divided into training set, validation set, and testing set without overlap. And the three sets are kept coincident for each trial of the five algorithms. The results are shown in Table 2, and the best results are emphasized in bold font.

Considering the training time, it is obvious that ELM is the fastest one because all the other four algorithms transfer ELM repeatedly. Besides, AIS-ELM's training time is slightly shorter than the other three methods because the times of ELM iteration are smaller than other three algorithms.

Then, focusing on the testing accuracy, it is easy to find that AIS-ELM has the highest mean testing accuracy in all the classification datasets. As for variance, AIS-ELM is the smallest in most instances and is slightly worse than the best one in a few cases. In addition, the good convergence property of clone selection algorithm shows that AIS-ELM outperforms the DSA-ELM, PSO-ELM, SaE-ELM, and ELM.

In addition, we have done the Wilcoxon's signed-rank test [31], and the W-value is 0, which is less than the critical value at p<=0.05. Therefore, the results show that AIS-ELM is significantly different from DS-ELM, PSO-ELM, SaE-ELM, and ELM, which indicates that AIS-ELM outperforms the other four approaches on eight classification datasets.

Besides, we compare the training time between AIS-ELM and BP, SVM, and traditional ELM on three benchmark classification problems. The results are shown in Table 3.

From Table 3, although AIS-ELM is slower than traditional ELM because of iterations, its training speed is significantly faster than that of BP and SVM.

### 5.2. Regression

In this subsection, the five algorithms are compared on the five regression benchmark problems. The five datasets are Breast Cancer, Parkinson, SinC, Servo, and Yacht Hydro (Yacht), respectively. Detailed description of the five datasets is shown in Table 4.

Attributes of all the datasets have been normalized to [-1, 1] and we focus on the training time and testing accuracy's means and variance. A 20-fold cross validation method is taken to get the average of 20 repeated experiments to minimize the error. The whole dataset is divided into training set, validation set, and testing set without overlap. The results are shown in Table 5.

Considering the training time, it is also obvious that traditional ELM is the fastest because of the same reason in 5.1. As for the testing accuracy, AIS-ELM, DS-ELM, PSO-ELM, and SaE-ELM obtain better results with less hidden nodes than ELM, which means that AIS-ELM, DS-ELM, PSO-ELM, and SaE-ELM can achieve better generalization performances with more compact networks. And the RMSE of AIS-ELM is smaller than other four algorithms. Therefore, it can be concluded that AIS-ELM can achieve better performance than other four algorithms on regression problems.

## 6. Conclusion

In this paper, first we introduce the standard ELM and artificial immune system; then we propose a new approach named artificial immune system extreme learning machine (AIS-ELM). In AIS-ELM, AIS is used to optimize the input weights through clone, mutation, and substitution process.

In the experiment part of this paper, AIS-ELM is compared with DS-ELM, PSO-ELM, SaE-ELM, and the traditional ELM on thirteen well-known benchmark datasets (eight classification datasets and five regression datasets) obtained from UCI Machine Learning Repository. Besides, the training times between AIS-ELM and BP, SVM, and traditional ELM are compared on three benchmark classification problems. Experimental results show that AIS-ELM can achieve better testing results (smaller RMSE on regression and higher accuracy on classification) than other DS-ELM, PSO-ELM, SaE-ELM, and the traditional ELM in most cases, and its training speed is significantly faster than that of BP and SVM. According to the global search ability [21] and good convergence [22] of AIS, our artificial immune system extreme learning machine is superior to the other methods both on the eight classification datasets and five regression datasets in the experiments. In addition, there are six medical datasets among the thirteen datasets, which can prove that AIS-ELM can also play an excellent role in healthcare. Future research works will be concentered on how to apply the current immune system to some new directions, such as NLP and computer vision.

## Figures and Tables

**Algorithm 1:**
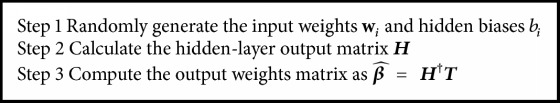
Standard ELM.

**Algorithm 2:**
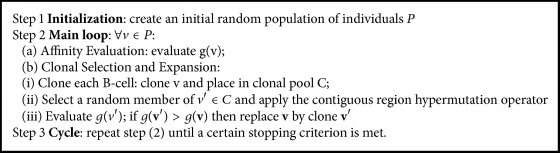
B cell algorithm.

**Algorithm 3:**
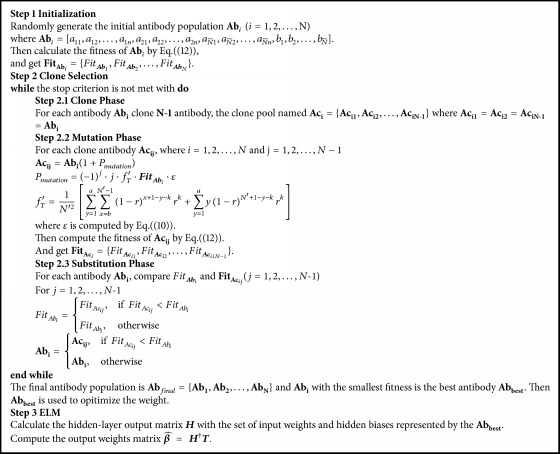
Artificial immune system extreme learning machine.

**Table 1:** Detailed description of the eight benchmark classification datasets

Dataset	Data			Attributes	Classes
	Training	Validation	Testing		
Ecoli	180	78	78	7	8
Diabetes	384	22	192	8	2
Epileptic Seizure	6000	2750	2750	179	5
Heart Disease	150	76	76	75	5
Iris	70	40	40	4	3
Glass	100	57	57	9	7
Image	1200	555	555	19	7
Satellite	3435	1500	1500	36	7

**Table 2:** Results of the five algorithms on eight benchmark classification datasets.

Dataset	Algorithm	Training	Testing Accuracy (%)		Hidden
		Time (s)	Means	StDev	Nodes
Ecoli	AIS-ELM	2.232	**87.087**	**1.32**	20
	DS-ELM	2.523	86.352	1.43	20
	PSO-ELM	2.257	86.623	1.67	20
	SaE-ELM	2.608	85.985	1.78	20
	ELM	0.003	84.678	2.12	30
Diabetes	AIS-ELM	2.865	**82.771**	0.67	20
	DS-ELM	3.192	80.667	0.65	20
	PSO-ELM	3.993	80.123	**0.53**	20
	SaE-ELM	4.216	81.673	0.76	20
	ELM	0.004	78.984	1.21	30
Epileptic	AIS-ELM	80.379	**83.412**	**0.78**	150
	DS-ELM	82.458	82.345	0.95	150
	PSO-ELM	84.912	81.627	1.12	150
	SaE-ELM	84.233	81.765	1.05	150
	ELM	3.976	80.026	1.21	180
Heart Disease	AIS-ELM	10.923	**80.234**	**1.53**	20
	DS-ELM	11.329	79.637	1.56	20
	PSO-ELM	10.993	78.942	1.73	20
	SaE-ELM	12.265	78.762	1.69	20
	ELM	0.013	76.149	1,96	30
Iris	AIS-ELM	1.0835	**96.921**	**0.35**	20
	DS-ELM	1.255	96.488	0.69	20
	PSO-ELM	1.1315	96.124	0.83	20
	SaE-ELM	1.362	95.642	0.74	20
	ELM	0.001	93.439	1.26	30
Glass	AIS-ELM	2.632	**68.453**	**1.67**	20
	DS-ELM	3.026	65.345	1.89	20
	PSO-ELM	3.067	65.438	1.91	20
	SaE-ELM	3.036	65.087	2.23	20
	ELM	0.003	60.267	2.12	30
Image	AIS-ELM	29.221	**94.55**	0.659	90
	DS-ELM	31.976	93.78	**0.528**	90
	PSO-ELM	32.103	93.23	1.014	90
	SaE-ELM	32.641	92.11	0.832	90
	ELM	0.0493	92.56	0.783	120
Satellite	AIS-ELM	37.424	**88.346**	**0.87**	100
	DS-ELM	39.856	87.265	0.97	100
	PSO-ELM	39.613	86.795	1.08	100
	SaE-ELM	40.238	86.715	0.96	100
	ELM	0.0624	85.028	0.99	150

**Table 3:** Results of the four algorithms on three benchmark classification datasets.

Algorithm	Satellite	Image	Epileptic Seizure
	Training Times (s)	Training Times (s)	Training Times (s)
AIS-ELM	37.424	29.221	80.379
SVM	129.235	103.496	339.648
BP	67.329	58.637	186.247
ELM	0.0624	0.0493	3.976

**Table 4:** Detailed description of the five benchmark regression datasets.

Dataset	Data			Attributes
	Training	Validation	Testing	
Breast Cancer	98	50	50	32
Parkinson	500	270	270	26
SinC	5000	2500	2500	1
Servo	384	192	192	4
Yacht Hydro	150	79	79	13

**Table 5:** Results of the five algorithms on the five benchmark regression datasets.

Dataset	Algorithm	Training	Testing Accuracy		Hidden
		Time (s)	Means	StDev	Nodes
Breast Cancer	AIS-ELM	14.898	**2.46E-01**	**1.32E-02**	30
	DS-ELM	15.367	2.53E-01	1.63E-02	30
	PSO-ELM	15.902	2.98E-01	2.35E-02	30
	SaE-ELM	16.342	2.65E-01	1.76E-02	30
	ELM	0.008	2.99E-01	2.07E-02	50
Parkinson	AIS-ELM	20.287	**1.68E-01**	**3.35E-02**	30
	DS-ELM	21.349	1.79E-01	3.67E-02	30
	PSO-ELM	22.547	1.87E-01	4.12E-02	30
	SaE-ELM	22.975	1.89E-01	3.95E-02	30
	ELM	0.011	2.12E-01	4.53E-02	50
Servo	AIS-ELM	14.942	**8.81E-02**	9.83E-03	20
	DS-ELM	15.256	9.41E-02	**9.63E-03**	20
	PSO-ELM	15.278	1.17E-01	1.35E-02	20
	SaE-ELM	15.456	1.07E-01	1.45E-02	20
	ELM	0.007	1.35E-01	1.95E-02	30
Yacht	AIS-ELM	13.478	**1.76E-01**	**4.32E-02**	20
	DS-ELM	13.755	1.87E-01	4.52E-02	20
	PSO-ELM	13.834	2.45E-01	5.63E-02	20
	SaE-ELM	14.292	2.23E-01	5.60E-02	20
	ELM	0.006	2.67E-01	8.43E-02	30
SinC	AIS-ELM	33.012	**6.12E-03**	**3.54E-04**	30
	DS-ELM	33.514	6.35E-03	4.32E-04	30
	PSO-ELM	33.095	7.46E-03	5.41E-04	30
	SaE-ELM	33.821	7.93E-03	5.76E-04	30
	ELM	0.013	8.01E-03	3.84E-04	50

## Data Availability

The classification datasets and regression datasets supporting the findings of this study are from previously reported studies and datasets, which have been cited. The processed data are available at UCI Machine Learning Repository [Online] (http://archive.ics.uci.edu/ml).
